# Effect of gonadotropin administration on reproductive performance in relation to the age at first conception, time of application, and body condition of weaned sow

**DOI:** 10.5194/aab-68-211-2025

**Published:** 2025-03-17

**Authors:** Marija Vogrin Bračič, Maja Prevolnik Povše, Janko Skok, Dejan Škorjanc

**Affiliations:** 1 Department of animal science, Faculty of Agriculture and Life Sciences, University of Maribor, Hoče 2311, Slovenia

## Abstract

The administration of hormone preparations to synchronize the onset of oestrus in gilts and sows is a good way of achieving breeding objectives economically on large-scale farms. Therefore, the objective of this study was to analyse the effects of the timing of administration of gonadotropins (400 IU (international unit) eCG – equine chorionic gonadotropin – and 200 IU hCG – human chorionic gonadotropin; Intervet International B.V.), the age of gilts at first conception, and their backfat thickness (BFT) at weaning on the length of weaning to oestrus interval (WEI) and litter size in the following four parities. A total of 578 crossbred sows ( Landrace×Large White ) from a commercial farm were divided into three groups: the control PG0 (without gonadotropins, n=192 ), PG72 (gonadotropin administration 72 h before weaning, n=192 ), and PG24 (gonadotropin administration 24 h after weaning, n=194 ). Within each group, gilts were also divided into two classes according to their age at first conception: 240–260 d and >260d . Body condition of sows was determined based on BFT, measured on the 110th day of gestation and at weaning on the 28th day of lactation, which were classified into four classes of body condition within each reproduction cycle. Primiparous sows that were younger at first conception (240–260 d and BFT >14.3mm at weaning) had a significantly shorter WEI but only at first weaning. At the subsequent farrows, age at first conception had no significant effect on WEI length. With subsequent farrows, increasing BFT along with gonadotropin administration (72 h before and 24 h after weaning) had a significant effect on reducing WEI. Age at first conception (240–260 d) of the primiparous sow with both gonadotropin administrations significantly increased the number of live-born piglets in all parities studied in comparison to the older group ( >260d ). In conclusion, the study showed that it is necessary to adjust the selective timing of gonadotropin administration at weaning to the age of primiparous sows and their body condition.

## 1 Introduction

Entry of gilts into herd reproduction and sows return to oestrus within 7 d of weaning are crucial for the success of reproductive performance in commercial herds. For both criteria i.e. expressed as entry to service interval and weaning to oestrus interval (WEI), shortening the interval means reducing non-productive days and increasing efficiency of the herd productivity.

In practice, we strive to involve the gilts in reproduction as early as possible, i.e. during puberty at about at least 5–6 months of age and with a body weight of approximately 90–100 kg (Čechová and Tvrdoñ, 2006; Manjarin et al., 2010; Martinat-Botté et al., 2011). At this time, gilts often do not yet have a regularly cyclic oestrus, and the external signs of being in heat are only weakly expressed or having silent periods of being in heat. In gilts, we can implement insemination at the second or third oestrus, which also increases the number of unproductive days. There is no common rule regarding the optimal age at first conception, which can vary considerably, i.e. <230d (Clowes et al., 2003; Hughes et al., 2010; Patterson et al., 2010), 240 and 260 d (Waehner, 2006; Heurich and Huehn, 2008; Young et al., 2008), or 260–280 d (Le Cozler et al., 1997).

Modern hybrid primiparous sows normally lose their body reserves during lactation. At the weaning, they consequently have insufficient body reserves to successfully begin the next reproductive cycle (Kemp and Soede, 2012). In this period, the primiparous sows meet with high metabolic demands due to nursing large litters; thus, feed intake of sows is often not sufficient to fulfil energy demands for their maintenance, growth, milk production, and subsequent fertility (Arend et al., 2023). High sow milk production requires the mobilization of body reserves during lactation (Quesnel et al., 2007). Therefore, adipose and skeletal muscle tissue undergoes catabolic processes, which are necessary for the synthesis of milk fats and proteins. It was found that the milk fat production was related to the back depth loss, while milk protein production was related to the loin muscle depth/thickness loss (Costermans et al., 2020a). To control and prevent fertility problems, the body condition of sows can be measured and evaluated by indicators such as backfat thickness (BFT). In primiparous sows, BFT at weaning has a strong influence on size of follicle diameter, weaning to ovulation interval, and weaning to oestrus interval (Pearodwong et al., 2020).

To avoid the problems mentioned, oestrus can be induced and synchronized in gilts and later at weaning of primiparous and multiparous sows. For this purpose, equine chorionic gonadotropin (eCG) could be used in sows, which has a similar effect as follicle-stimulating hormone (FSH) and human chorionic gonadotropin (hCG), which is similar to luteinizing hormone (LH) (Waehner, 2006; Bruessow et al., 2009). Gonadotropin-realizing hormone (GnRH) is secreted by the hypothalamus and stimulates secretion of FSH and LH hormones of the pituitary gland (Waehner and Richter, 2011; Harris et al., 2012).

For stimulation of gilts and primiparous sows, physical or fence line boar exposure in combination with the administration of gonadotropins can be applied (Willenburg et al., 2003; Bartlett et al., 2009; Wettere et al., 2013; Lewchalermwong et al., 2020; Knox et al., 2021). There are also several options for administration at this stage. Animals can be treated with only 600 IU of equine chorionic gonadotropin (eCG) (Manjarin et al., 2009); with a combination of 400 IU equine chorionic gonadotropin (eCG) and 200 IU human chorionic gonadotropin (hCG) (PG600) applied before, at, or after weaning (Kirkwood, 1999; De Rensis et al., 2003; Breen et al., 2006; Vargas et al., 2006); with 1.5 and 2 doses of PG600 (Breen et al., 2006); or with PG600 with an additional 200 IU hCG injected at different times (Manjarin et al., 2010, 2020).

When using hormonal synchronization, a very important parameter is time of gonadotropin application. Well-known methods are the administration of gonadotropins 48 h before weaning, which shortens WEI and slightly enlarges the size of the next litter (De Rensis et al., 2003), or administration on the day of weaning, where no advantage of gonadotropin usage was determined (Bates et al., 2000) or shortening of WEI has been stated (Knox et al., 2001; Bennett-Steward et al., 2008). Similarly, application 24 h after weaning shortens WEI (Vargas et al., 2006; Škorjanc et al., 2008) and enlarges the litter size (Vargas et al., 2006; Waehner, 2006; Waehner and Richter, 2011).

Thicker backfat at weaning was moderately but significantly correlated with lower loss of BFT during lactation in the primiparous sows (Škorjanc et al., 2008). Reduced BFT loss during lactation is associated with shortening of the unproductive phase – WEI (Koketsu, 1999; Clowes et al., 2003; Eissen et al., 2003). A larger second litter size was affected by absolute body reserves at weaning (Kongsted and Hermansen, 2009; Schenkel et al., 2010), where the sow has at least 16 mm of BFT at weaning (Schenkel et al., 2010). Return to oestrus after weaning depends on metabolic state of the sow (Foxcroft, 2012). Optimization of the WEI of primiparous sows and their litter size requires alteration of the age at first conception and suitability of body condition, based on the control of body fat reserves that had been estimated by BFT during the lactation period (Dourmad et al., 2001; Galan et al., 2007; Rossi et al., 2008; Tummaruk, 2013). Selection of sows based on lower age at first conception leads to shortening of WEI and enlargement of litter size (Imboonta et al., 2007).

The objective of this study was to investigate the effects of age of gilts at first conception, backfat thickness of primiparous sows at weaning, and different timings of gonadotropin administration on weaning to oestrus interval and subsequent litter size. In addition, we extended the study to the effects of gonadotropin administration on the next parities to determine during which subsequent parities and under which prerequisite body condition is the administration of gonadotropins useful to achieve optimal results in sow reproduction.

## 2 Material and methods

### 2.1 Animals

The study was performed in a highly productive commercial herd in the eastern part of Slovenia over a period of 4 years, and it was carried out respecting the legislation on animal protection (EU Council Directive 2008/120/EC, 2008).

A total of 578 primiparous crossbreed Landrace × Large White gilts ( L×LW ) was recorded. Gilts were further divided into two groups based on age at first conception: 240–260 d and over 260 d. The data were collected over three full reproductive/lactation cycles to the beginning of the fourth.

### 2.2 Management

#### 2.2.1 Housing

From the confirming of gestation on the 28th day after artificial insemination until the 109–112th day of gestation, sows were housed in the gestation building in group pens of 3.6m×4.8m size for seven sows (i.e. $2.5\;m^{2}$ per sow). The area for resting ( ≈2/3 of the pen) had a solid floor and solid sidewalls. The remaining part ( ≈1/3 of the pen) had a slatted floor and latticed walls that were intended for urination and excretion. In this part of the barn, an average temperature of 18.5 °C and illumination of 60 lux were maintained.

Up to 1 week prior to expected delivery, sows were moved to the farrowing building where they stayed until the end of lactation. Farrowing was synchronized to the 114th day of gravidity using 0.7 mL prostaglandin $F_{2\alpha }$ (Estrumate^®^, Intervet International B.V., the Netherlands). Sows were housed in the individual farrowing crates of $3.8\;m^{2}$ with partly slatted floor ( ≈2/3 of pen) and solid sidewalls. In the farrowing building, average temperature and illumination were 19.0 °C and 60 lux, respectively. Near the sow's head, there was a special place of $0.65\;m^{2}$ for piglets, which was equipped with an electric floor heating area and an additional infrared electric bulb to assure optimal temperature for piglets.

From weaning to the 28th day of gestation, sows were kept in the mating building in the individual pens of an area of $1.6\;m^{2}$ ( 2.3m×0.7m ) with slatted floor and lattice walls. Average temperature in the mating house was 19.5 °C . A 16 h illumination of 330 lux was assured until the insemination, and it was then decreased to 60 lux. After the 28th day, pregnant sows were moved back to the group pens.

#### 2.2.2 Feeding

Throughout the experiment, the animals were fed using an automatic feeding system. The frequency of feeding and type of feeding mixture were adapted to the phase of reproductive cycle of animals. Sows had ad libitum access to water through the automatic nipple water supply system.

In the period of gestation, pregnant sows were fed once a day (in the morning) with a commercial pelleted feeding mixture for sows. The mixture contained 12.1 $\mathrm{MJ}\;{\mathrm{kg}}^{-1}$ metabolizable energy (ME), 13.0 % crude protein (CP), and 0.6 % lysine (LYS). In the period from the 28th to the 84th day of gestation, sows daily consumed 25–43 and 43–50 MJ ME in the last month of gestation (84th to 112th day). Since the 113th day of gestation, a complete feeding mixture for lactating sows was used, which contained 13.1 $\mathrm{MJ}\;{\mathrm{kg}}^{-1}$ ME, 16.2 % CP, and 0.9 % LYS. Average daily feed intake decreased from 26.0 MJ ME on the 113th day to 13.0 MJ ME on the 115th day.

During lactation, feeding was carried out twice a day in the first 2 weeks after farrowing and four times daily in the second half of lactation. In the same periods, daily feed intake amounted to 65.0 and 84.5 MJ ME, respectively. Three days before weaning, the amount of offered feeding mixture was reduced, resulting in a decline in daily feed intake to 13.0 MJ ME on the day before weaning. Since the fourth day after birth, piglets were offered a special pelleted feeding mixture (14.2 $\mathrm{MJ}\;{\mathrm{kg}}^{-1}$ ME, 20.5 % CP, and 8.1 % crude fat) and had ad libitum access to water.

From weaning to artificial insemination, sows were fed once a day using a feeding mixture for pregnant sows (daily feed intake of 46 MJ ME). Additionally, 150 g of fish meal (containing 72 % CP, 150 g of dextrose, and 10 g of monocalcium phosphate (DK-03-3-FM-001, Denmark)) and 4 g of a vitamin–mineral mixture (containing vitamin E and selenium; Solvimin^®^ Selen, Krka d.d., Novo mesto, Slovenia) were added daily per sow.

### 2.3 Measurements of backfat thickness and body condition

Measurements of backfat thickness (BFT) were recorded on the 110th day of gestation and at weaning (28th day of lactation) using the Dramiński ultrasound apparatus (ultrasound scanner Animal Profi, Dramiński, Poland). Backfat thickness was measured at three different points (ehner et al., 2001): at the area of the last rib, i.e. 7 cm lateral from the spinous process (*processus spinosus*), and 15 cm cranial and 15 cm caudal from the first measurement.

### 2.4 Application of gonadotropins

To stimulate oestrus and ovulation in primiparous sows, a PG600^®^ (Intervet International B.V., the Netherlands) was used containing equine (eCG) and human chorionic gonadotropins (hCG). A 5 mL dose of PG600^®^ (400 IU of eCG, 200 IU of hCG) was injected into the cervical region of sows. Regarding the time of administration of PG600, we formed three groups of animals: without gonadotropin (PG0, n=192 ), gonadotropin administration 24 h after weaning (PG24; n=194 ), and gonadotropin administration 72 h before weaning (PG72; n=192 ). During the experiment, there were several batches of 50–80 sows that were weaned at the same time. The interval between the individual batches was approximately 1 week. Within each batch, the sows were randomly divided into three groups for the application of PG600.

### 2.5 Oestrus detection and insemination

After weaning, the occurrence of oestrus was checked twice a day (in the morning and evening). Sows were exposed to boars by a fence line for 15 min. The sows were artificially inseminated twice. The first artificial insemination was carried out within 12 h of the first signs of oestrus and a second after 12 h. Artificial insemination was carried out in the presence of a boar with a 100 mL dose containing $3.0\times 10^{9}$ sperm cells (Sacco bag, Medi Chimica International, Italy) and addition of 1 mL of oxytocin (Intervet International B.V., the Netherlands) according to Waehner (2006). Success of insemination was checked after 28 d using an ultrasound apparatus (Agroscan, ECM, France). Non-pregnant animals went again through repeated reproduction cycles or were culled.

### 2.6 Reproduction traits

The main indicators used to evaluate reproductive performance were the length of weaning to oestrus interval (WEI, in days) and litter size (i.e. number of live-born piglets) in the next parity. Reproduction characteristics were monitored during three parities to the beginning of the fourth.

### 2.7 Statistical analysis

Statistical analysis was carried using IBM SPSS Statistics version 21. Pearson's correlation coefficients ( r ) of WEI and litter size with different body measurements of BFT were calculated. The BFT at weaning (Table 1) was used as a measure of body condition.

**Table 1:** Pearson's correlation coefficients of WEI and litter size with backfat thickness (BFT) measurements in the first three parities.

n=578	Parity 1		Parity 2		Parity 3	
	WEI^1^	Litter size^2^	WEI^1^	Litter size^2^	WEI^1^	Litter size^2^
Backfat thickness						
At farrowing	-0.28	0.20	-0.50	0.37	-0.51	0.33
At weaning	-0.28	0.23	-0.49	0.37	-0.53	0.34
Loss during lactation	0.26	-0.24	0.37	-0.29	0.52	-0.31

^1^ WEI – weaning to oestrus interval; ^2^ Litter size – number of piglets born alive.

For each parity, values of this trait were divided into four body condition classes or scores with quartile values being boundaries/limits between classes (Table 2).

**Table 2:** Determination of sow body condition based on their BFT measurements.

Body condition	BFT, mm		
	Parity 1	Parity 2	Parity 3
1	<12.0	<12.3	<15.0
2	12.0–14.2	12.3–15.6	15.0–18.6
3	14.3–16.7	15.7–19.0	18.7–21.3
4	>16.7	>19.0	>21.3

The effect of gonadotropin application (PG0, PG24, PG72), age at first conception (240–260 d and >260d ), and body condition (1–4) were assessed using analysis of variance (generalized linear model (GLM) procedure) and the following model:

1 $$\begin{array}{rl} Y_{\mathrm{ijkl}} & =\mu +G_{i}+C_{j}+A_{k}+(G\times C{)}_{\mathrm{ij}}+(G\times A{)}_{\mathrm{ik}}\\ & +(C\times A{)}_{\mathrm{jk}}+(G\times C\times A{)}_{\mathrm{ijk}}+e_{\mathrm{ijkl}}, \end{array}$$

where $Y_{\mathrm{ijkl}}$ represents the studied trait (WEI or litter size); μ represents mean; $G_{i}$ represents effect of PG treatment (three-level variable: PG0 (control), PG24, PG72); $C_{j}$ represents effect of body condition (four-level variable: score 1–4 based on BFT); $A_{k}$ represents effect of age at first conception (two-level variable: 240–260 d, >260d ); $(G\times A{)}_{\mathrm{ij}}$ represents effect of interaction between PG treatment and age at first conception; $(G\times C{)}_{\mathrm{ik}}$ represents effect of interaction between PG treatment and body condition; $(C\times A{)}_{\mathrm{jk}}$ represents effect of interaction between body condition and age at first conception; $(G\times C\times A{)}_{\mathrm{ijk}}$ represents the effect of triple interaction among PG treatment, body condition, and age at first conception; and $e_{\mathrm{ijkl}}$ represents random error. In the case of statistically significant triple interaction among studied effects, the analysis was performed separately according to the age of first conception using the model with fixed effects of PG treatment and body condition as well as their interaction. Mean values were compared using Tukey's honestly significant difference (HSD) post hoc test.

## 3 Results

### 3.1 Effect of gonadotropin (PG) treatment, body condition, and age at first conception

As presented in Table 3, WEI was particularly affected by PG treatment, body condition, and their interaction. Other factors were insignificant, with the exception of the age of the sows.

**Table 3:** Summary of the significance of studied factors and interactions on WEI and litter size ( p -values).

	WEI^1^			Litter size^2^		
Effect	Parity 1	Parity 2	Parity 3	Parity 1	Parity 2	Parity 3
PG treatment	<0.0001	<0.0001	<0.0001	0.1766	<0.0001	<0.0001
Body condition	<0.0001	<0.0001	<0.0001	<0.0001	<0.0001	<0.0001
Age at first conception	0.0005	0.6070	0.5613	<0.0001	<0.0001	<0.0001
PG treatment × body condition	0.0001	<0.0001	<0.0001	0.8869	0.0290	0.0317
PG treatment × age at first conception	0.7959	0.4970	0.9720	0.0525	0.0208	<0.0001
Body condition × age at first conception	0.6781	0.4708	0.9537	0.0270	0.0001	0.0001
PG treatment × body condition × age at first conception	0.2953	0.2838	0.0672	0.5082	0.0029	0.0172

^1^ WEI – weaning to oestrus interval; ^2^ Litter size – number of live-born piglets in the next parity.

The number of live-born piglets at first farrowing is significantly affected by gonadotropin administration, age at first conception, and the interaction between these factors (Table 4). Table 4 shows that the group of primiparous sows without treatment with gonadotropins (control) had a significantly larger litter than the group with gonadotropins administered ( 12.0±0.1 , 10.7±0.1 ; P<0.0001 ). The age of the gilts at the time of first conception also had a significant influence ( P<0.0001 ). Younger gilts (240–260 d) had significantly larger litters than older gilts at first conception ( 12.3±0.1 , 10.9±0.1 ). A statistically significant greater loss of BFT at the end of lactation was observed in primiparous sows that were not treated with gonadotropins ( 4.9±0.1 , 4.6±0.1 ; P<0.0383 ). No significant differences in body weight at weaning were observed between the groups of sows studied.

**Table 4:** The effect of gonadotropin treatment, age at first conception, and their interaction on litter size at first farrowing and BFT.

	With	Without	Age (d)		Effects		
	PG600	PG600	240–260	>260	PG600	Age	PG600 × Age
Farrowing							
No. of live-born piglets	10.7±0.1	12.0±0.1	12.3±0.1	10.9±0.1	<0.0001	<0.0001	0.0003
BFT, mm	19.2±0.2	19.0±0.2	19.0±0.2	19.1±0.2	0.2621	0.4759	0.1755
Weaning							
BFT, mm	14.6±0.2	14.1±0.2	14.1±0.2	14.5±0.2	0.1265	0.2185	0.2799
BFT loss, mm	4.6±0.1	4.9±0.1	4.6±0.1	4.6±0.1	0.0383	0.0526	0.6053
Weight, kg	170.2±1.0	172.0±0.9	169.8±0.9	172.4±1.0	0.1861	0.0510	0.3520

**Figure 1:**
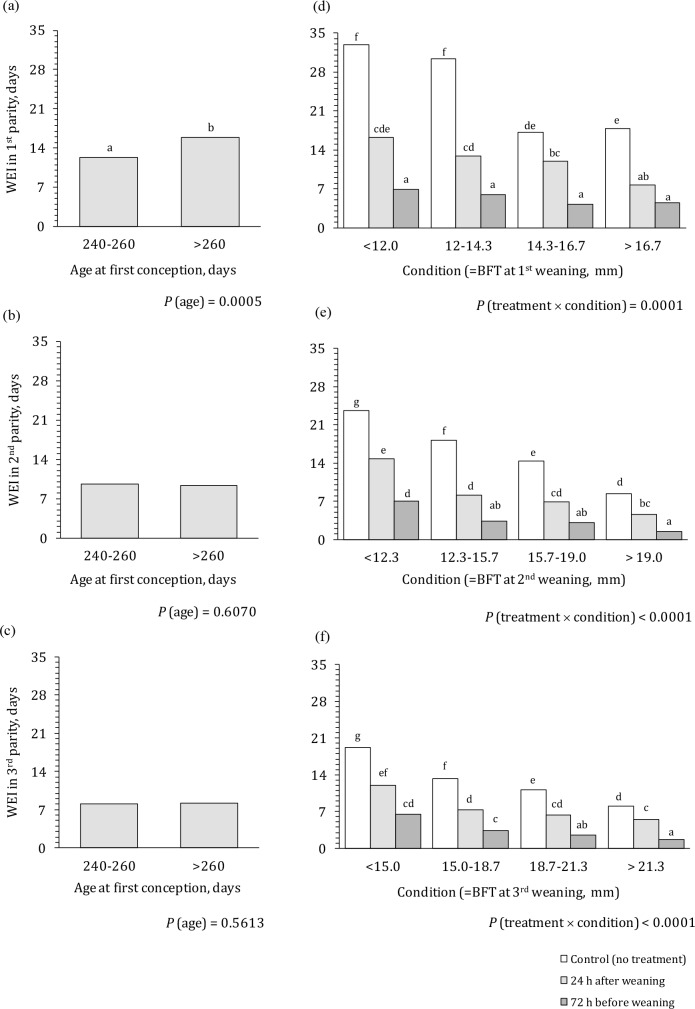
Effect of treatment, age at first conception, and body condition on WEI in the first three parities. WEI – weaning to oestrus interval; BFT – backfat thickness; a–g means different letters differ significantly ( P<0.05 ).

The effect of PG treatment, body condition, and age at first conception on WEI in the first three parities is presented in Fig. 1. Age at first conception (Fig. 1a–c), which did not interact with any other factor, showed a significant effect on WEI only in the first parity. Primiparous sows that were over 260 d old when initially fertilized had significantly longer WEI compared to younger sows (15.9 vs. 12.3 d, respectively). In the subsequent parities, the effect of age at first conception was no longer detected.

A highly significant effect of PG treatment, body condition, and their interaction on WEI was observed in all three parities (Fig. 1d–f). Untreated primiparous sows (Fig. 1d) in good body condition (scores 3 and 4) had a half shorter WEI compared to sows in poor body condition (scores 1 and 2; i.e. 32 vs. 17 d, respectively). Irrespective of body condition score, PG treatment significantly shortened WEI, especially PG72 treatment where WEI was 75 %–80 % shorter compared to the control group from 30–33 to 6–7 d for sows in poor body condition (score 1 or 2) and from 17–18 to 4–5 d for sows in good body condition (score 3 or 4). Application of PG24 was less effective; it mainly shortened WEI for 50 %–60 % compared to the untreated sows. In the case when gonadotropins were used, the effect of body condition on WEI was decreased (from 16.3 to 7.7 d, when stimulating sows 24 h after weaning) or even absent (when stimulating sows 72 h before weaning). Untreated primiparous sows in good body condition (scores 3 and 4) reached the same result (i.e. WEI of 17–18 d) as sows in poor body condition (1, 2) treated with PG24. However, WEI length obtained when using PG72 could not be achieved with good body condition only.

In the second and third parities (Fig. 1e and f), similar patterns could be observed with absolute values for WEI being noticeability lower compared to the WEI in the first parity. The strongest effect was caused by PG72 (65 %–85 %, shortening WEI in different body condition classes). Application of PG24 again showed an intermediate effect. In the control group, WEI uniformly decreases with increasing body condition (for 55 %–65 %); WEI was also shortened with increased body condition when gonadotropins were applied. With improved body condition only (without hormonal stimulation), it was possible to reach WEI of 8.4 and 8.1 d (body condition score 4) in the second and third parities, respectively. These values were comparable to WEI of sows in a poor body condition (score 1) treated with PG72 or to sows in a medium body condition (score 2, 3) treated with PG24. However, for sows in good body condition (score 4), WEI could be significantly shortened further by about 7.0 d (to 1.4 and 1.7 d in second and third parities, respectively) with application of PG72.

### 3.2 Effect of gonadotropin (PG) treatment, body condition, and age at first conception on litter size

**Figure 2:**
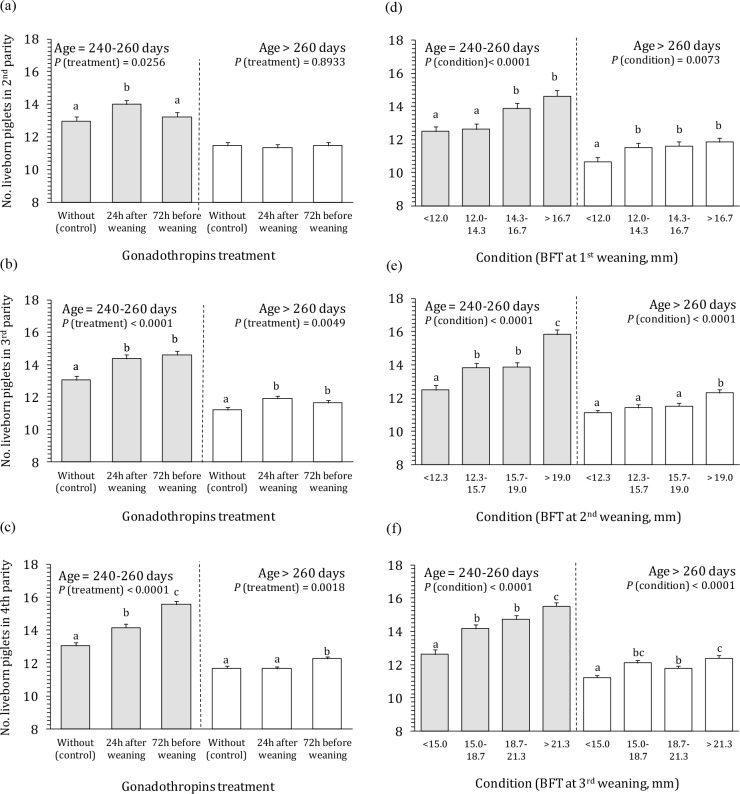
Effect of gonadotropin treatment and body condition on the number of live-born piglets in the second to fourth parities according to the age at first conception. 1 number of live-born piglets in the next parity; BFT – backfat thickness; a–h means different letters differ significantly ( P<0.05 ); the effect of interaction is mainly insignificant.

For litter size, more complex (co)influence of studied factors was found compared to WEI. Main part of factors and interactions including the triple interaction significantly affected litter size (Table 3 – analysis of variance). For this reason, data analysis and presentation were performed separately according to the age of first conception (for sows successfully inseminated at lower and higher age, i.e. 240–260 and >260d , respectively). The effects of treatment, body condition, and age at first conception on the litter size in the second, third, and fourth parities are presented in Fig. 2. In general, substantially smaller litter size (up to 3.5 piglets) was observed for older sows irrespective of PG treatment and body condition. Moreover, the effects of PG treatment and body condition were considerably less pronounced in older sows.

Beneficial effect of both PG applications on litter size was noted in younger sows (Fig. 2a–c). The PG24 caused increase of litter size for about 1 piglet compared to the control in all three parities. Similar result was obtained also for PG72 in the third parity. In the other hand, PG72 showed no effect in the second parity and especially strong effect ( +2.5 piglets) in the fourth parity. In older sows at first conception, hormonal stimulation had no effect on litter size of second parity sows. In the next parities of this group, it caused enlargement for a maximum of 0.7 piglets.

Litter size was significantly affected also by body condition (Fig. 2d–f), especially in younger sows. Improvement/increase of body condition (from 1 to 4) enlarged litter size of for 2.1–3.4 piglets in younger sow, and ≈1.2 piglets in litters of older sows, respectively.

## 4 Discussion

The current study demonstrated the importance of backfat thickness at weaning of primiparous sows for proper timing and administration of gonadotropins to shorten the weaning to oestrus interval (WEI) and enlarge subsequent litter size. However, the age of the gilts at first conception also plays a very important role in the reproductive success of the herd.

The introduction of gilts to the herd is often accompanied by the problem of undeveloped or poorly developed signs of oestrus. Therefore, on commercial farms, induction of oestrus in gilts is achieved by exposing gilts to boars together with administration of gonadotropins (Knox et al., 2021). Treatment of gilts with gonadotropins PG600 at 170 d of age induced signs of oestrus on day six, but the results suggest a probable defect in the expression of oestrus due to follicular variability in oestrogen production or response at the level of the hypothalamus to so-called oestrogen feedback (Lewchalermwong et al., 2020). Problems with variability in hormonal function of gilts' follicles after gonadotropin administration are associated with smaller litter size at first farrowing. For example, gilts weighing 85–90 kg treated with PG600 and artificially inseminated between days 4 and 5 had a smaller litter at first farrowing (Holtz et al., 1999).

The lower loss of backfat thickness may be attributed to the fact that this group also had a smaller litter size. A smaller first litter means that they also completed the first lactation in a probably better body condition with more body reserves. Sows with large litters have greater losses in body weight and backfat thickness until weaning (Arend et al., 2023). Large litters require more mobilization of body reserves to produce the milk necessary for piglet growth, and the sow adapts to this situation with altered metabolism in response to the interaction between body reserves, nutrient intake, and food requirements (Quesnel et al., 2007). Body reserves are withdrawn so that body fat is used to form fat in milk, and body protein is used to synthesize milk proteins in the sow's milk (Costermans et al., 2020a). Consequently, a negative energy balance in the metabolism of primiparous sows impairs ovarian activity at weaning (Quesnel et al., 2007; Kauffold et al., 2008; Costermans et al., 2020b). Moreover, the weight loss during lactation in primiparous sows is associated with a lower number of embryo implantation sites, lower embryo survival, and lower embryo viability (Hoving et al., 2012). Embryo survival is the limiting factor for litter size at second parity (Wills et al., 2003). It has been reported that primiparous sows with a backfat thickness (BFT) of at least 16 mm at weaning had a significantly larger litter at the second parity (Schenkel et al., 2010).

The present study has shown that younger gilts (240–260 d) have a significantly shorter WEI than older gilts ( >260d ) at first conception, with body condition playing an important role regarding the length of WEI. A backfat thickness of at least 14.3 mm (body condition 3 and 4) is associated with a significantly shorter WEI compared to sows estimating a body condition score of 1 and 2 (approximately 33 d and 16 d, respectively). Previous studies have also found that thinner backfat at weaning reduced reproductive success (Kongsted and Hermansen, 2009), but lower age at first conception had a positive effect on litter size at subsequent parity and reduction of WEI (Wittmann, 2006; Imboonta et al., 2007). On the contrary, it was observed that the next litter size decreased when the WEI is longer than 3 d (Poleze et al., 2006), 4.5 d (Humpolicek et al., 2012), or 10 d (Marois et al., 2000). For example, the average size of the next litter decreases by 0.71 piglets when WEI is longer than 4 d (Karvelienė et al., 2008).

The weaning to oestrus interval can also be shortened by administering gonadotropins at different times, namely 48 h before weaning (De Rensis et al., 2003), on the day of weaning (Bates et al., 2000; Knox et al., 2001; De Rensis et al., 2003; Bennett-Steward et al., 2008; Cassar et al., 2010), or 24 h after weaning (Vargas et al., 2006). Application of gonadotropin 48 h before or at weaning did not affect the size of the next litter (De Rensis et al., 2003; Bates et al., 2000; Knox et al., 2001), but it was accompanied by a 4.5 d long WEI (De Rensis et al., 2003). On the contrary, it has been reported that administration of gonadotropin 24 h after weaning significantly affects the size of next litter (Vargas et al., 2006). Likewise, in our study, the application of gonadotropins 24 h after weaning had a positive effect on the increase in the number of piglets born alive in the second litter to 14.0, which is about 0.8 piglets more than in the application of gonadotropins 72 h before weaning or control. A more obvious effect of gonadotropin action is noticed at weaning at third parity, where there are no significant differences observed between application times of gonadotropin, but both gonadotropin groups have significantly larger litters (14.4, 14.6 live-born piglets) than the control group (13.1 live-born piglets, P<0.001 ). Perhaps one of the important findings of the present research is that the timing of gonadotropin administration is necessary to adapt to successive farrows. It appears that a more selective approach is needed at the end of the third lactation of multiparous sows. The multiparous sows given gonadotropin 72 h before third weaning had significantly increased the size of subsequent litters than gonadotropin given to sows 24 h after weaning in comparison to the control. The described results are only related for the group of gilts 240–260 d of age at first conception. Our data suggest that gilts that enter the reproductive cycle younger are more fertile and produced more piglets over the investigated parities. The results of this group are consistent with previous findings. Gilts that entered puberty at a younger age (240–260 d) produced more piglets in a period over three parities than older ones (260–280 d and/or >280d ) (Young et al., 2008). In addition, the results of the current experiment indicate that the fertility of younger gilts is much more under the effect of administered gonadotropins in terms of larger litter size in the subsequent second to fourth parities. These effects are not observed in the group of older selected gilts ( >260d ) at first conception. Generally, in the second to fourth parities studied in the older group, about 11–12 piglets are live-born, regardless of the method of administration and successive farrows or thickness of backfat.

There are several possible reasons that could contribute to the poorer response in the older group in the present study, but this cannot be generalized to all animals in the study group. Sows with low birth weight ( <1.0 to 1.28 kg) have a long-term negative effect on the subsequent rearing of piglets in the first three lactations. For instance, sows with low birth weight of less than 1 kg raise 4.5 fewer piglets (Magnabosco et al., 2016). In addition, gilts born to primiparous sows have lower backfat thickness at 100 kg of body weight and are older at first mating than gilts born from multiparous sows (Tummaruk et al., 2000). Another possible explanation could be related to the backfat thickness loss during first lactation in the present study. The gilts without administration with gonadotropins lost significantly more body reserves in the first lactation period due to larger first litter. More piglets in the litter means more milk production by the sow per day and higher levels of sow metabolism. However, milk production also depends on whether the teats were suckled in the previous lactation. Teats that were suckled in the first lactation produce more milk and develop better in the following second lactation than teats that were not suckled (Farmer et al., 2012).

## 5 Conclusions

We conclude from the present data that the age of the gilts (240–260 d) at first conception has a significant influence on the shortening of WEI only at first farrowing. In the subsequent farrows, this was not observed compared to the older group of gilts at first conception ( >260d ). Regardless of subsequent farrows and sow body condition, administration of gonadotropins 72 h before weaning had a significantly greater effect on shortening WEI by 65 %–88 %, depending on subsequent farrows and sow body condition at weaning (first >14.3mm , second >19.0mm , and third >21.3mm ). The age of the sows at first conception also has a strong influence on litter size, especially at the second to fourth farrows. In the group of older sows, we observed smaller litters of 3.5 piglets, regardless of the PG treatment and their body condition. The positive effect was observed in the group of younger sows treated 72 h after weaning, and it is particularly pronounced in the third and fourth farrows ( +2.5 piglets) compared to older sows. Therefore, we conclude and recommend that the administration of gonadotropins 72 h before weaning should be carried out in sows that were between 240 and 260 d old at the time of first conception and have a BFT mentioned above at the time of weaning. These body conditions must be met to achieve the maximum rearing effect in terms of reducing post-weaning unproductive days (WEI) and increasing the size of the next litter.

## Data Availability

The datasets used and/or analysed during the current study are available from the corresponding author on reasonable request.
